# The Paucity of Typical Pathology: An Autopsy Series of Typhus Group Rickettsiosis-Associated Hemophagocytic Lymphohistiocytosis

**DOI:** 10.3390/pathogens15020230

**Published:** 2026-02-19

**Authors:** Joshua Klinnert, Vasily Ovechko, Michelle M. Felicella, April McDougal, Sarah E. Muir, Patricia A. Crocquet-Valdes, David H. Walker, Lucas S. Blanton

**Affiliations:** 1 Department of Internal Medicine, University of Texas Medical Branch, 301 University Boulevard, Galveston, TX 77555, USA; jaklinne@utmb.edu; 2 Department of Pathology, University of Texas Medical Branch, 301 University Boulevard, Galveston, TX 77555, USApavaldes@utmb.edu (P.A.C.-V.); dwalker@utmb.edu (D.H.W.); 3 Division of Infectious Diseases, Department of Internal Medicine, University of Texas Medical Branch, 301 University Boulevard, Galveston, TX 77555, USA; 4 John Sealy School of Medicine, University of Texas Medical Branch, 301 University Boulevard, Galveston, TX 77555, USA; semuir@utmb.edu

**Keywords:** *Rickettsia typhi*, murine typhus, flea-borne typhus, endemic typhus, typhus group rickettsioses, hemophagocytic lymphohistiocytosis, autopsy, immunohistochemistry

## Abstract

Murine typhus (also called flea-borne or endemic typhus) is an undifferentiated febrile illness caused by the bacterium *Rickettsia typhi*. The disease, transmitted by rat and cat fleas, is endemic to seaboard regions worldwide. Recently, murine typhus has reemerged as an increasingly recognized cause of febrile illness in the United States, especially in Texas and Southern California. In addition to fever, manifestations often include headache, malaise, myalgias, and a maculopapular rash in approximately half of cases. Although usually considered a mild illness, when untreated, symptoms can last up to 3 weeks. Severe manifestations such as pneumonitis, acute kidney injury, and meningoencephalitis may occur. Historically, death has occurred in 0.4%, but in Southern California, the case fatality rate has been recently recorded at 1.8%. As murine typhus has reemerged, there have been growing reports that this infection has triggered hemophagocytic lymphohistiocytosis, a life-threatening hyperinflammatory syndrome. We herein report two fatal cases of hemophagocytic lymphohistiocytosis secondary to murine typhus. Autopsy revealed typhus group rickettsial antigen in tissues via immunohistochemistry, along with hemophagocytosis. Interestingly, the classic vascular and perivascular histopathologic findings associated with disseminated rickettsial infection were absent. These findings highlight an aberrant inflammatory cascade leading to hemophagocytic lymphohistiocytosis.

## 1. Introduction

Murine typhus is a flea-borne illness caused by *Rickettsia typhi*, an obligately intracellular Gram-negative bacterium [1]. *Rickettsia typhi*, along with the agent of louse-borne epidemic typhus (*Rickettsia prowazekii*), are typhus group rickettsiae, a phylogenetic subgroup that is separated from organisms of the spotted fever (e.g., *Rickettsia rickettsii*) and transitional groups (e.g., *Rickettsia akari*) [2]. Murine typhus is endemic to tropical and subtropical regions throughout the world and is especially prevalent along coastal areas, where it is maintained by rats (*Rattus ratus* and *R. norvegicus*) and transmitted by the rat flea (*Xenopsylla cheopis*). In North America, an alternate transmission cycle exists and involves opossums (*Didelphis virginiana*) and cat fleas (*Ctenocephalides felis*) as the amplifying reservoir and vector, respectively [3]. This latter ecologic cycle seems to be responsible for cases in southern California and Texas [4,5,6]. Murine typhus presents as an acute undifferentiated febrile illness and is often accompanied by headache, malaise, and myalgias. Rash, usually macular, occurs in about half of patients [7,8]. Severe manifestations, such as acute kidney injury (AKI), pneumonitis, and meningoencephalitis can occur [8]. The historic case fatality rate is only 0.4% [9,10], but in 2022, the case fatality rate in Los Angeles County, California has been reported to be as high as 1.8% [11]. More recently, along with the burgeoning cases being described in California and Texas, a life-threatening hyperinflammatory syndrome known as hemophagocytic lymphohistiocytosis (HLH) has been reported in association with murine typhus. HLH in association with murine typhus has been described in France, Spain, Tunisia, Mexico, and the United States (California and Texas) [12]. We herein report two fatal cases of HLH secondary to murine typhus. Autopsy revealed typhus group rickettsial antigen in tissues, along with hemophagocytosis. Interestingly, the classic vascular and perivascular histopathologic findings associated with disseminated rickettsial infection were absent. These findings may highlight an aberrant inflammatory cascade leading to HLH.

## 2. Case 1

A 69-year-old male was admitted from an outside emergency department with profound thrombocytopenia after presenting there with a chief complaint of fever. His symptoms started 4 days prior with fatigue and anorexia. He later developed fever and confusion as his illness progressed. Associated symptoms included hematuria and easy bruising. He otherwise denied dysuria, hemoptysis, hematochezia, melena, nausea, vomiting, diarrhea, dyspnea, or cough. His medical history included chronic obstructive pulmonary disease and hypertension. He had a 40 pack-year smoking history and consumed approximately six cans of beer daily. He lived in a suburban community of southeast Texas. On admission his vital signs were normal, and the reminder of his exam was remarkable only for bilateral upper extremity ecchymoses. Initial laboratory values demonstrated pancytopenia, elevated hepatic transaminases, and hyponatremia (Table 1). Other laboratory workup included normal serum B12, folate, prothrombin time, partial thromboplastin time, and direct antiglobulin testing. HIV and HCV tests were also negative.

On hospital day 2, fever to 39.6 °C was noted, the patient developed dyspnea, and a chest radiograph revealed a possible right basilar infiltrate. Computed tomography of the abdomen demonstrated splenomegaly (15 cm) and signs of hepatic steatosis. Hematology was consulted for worsening thrombocytopenia, and the patient was initiated on intravenous immunoglobulin and dexamethasone for concern for immune thrombocytopenic purpura. On hospital day 3, an increase in ferritin (3160 ng/mL) was noted, and HLH was suspected. Serum was sent for soluble IL2-R, and a bone marrow biopsy was performed. On hospital day 4, the patient required transfer to the medical intensive care unit where he was intubated for respiratory failure. His course was complicated by hypotension requiring use of norepinephrine and AKI necessitating continuous renal replacement therapy (CRRT).

**Figure 1:**
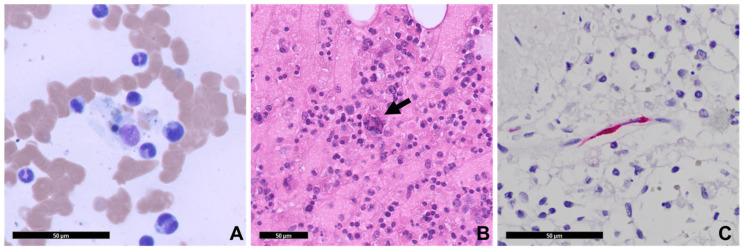
Case 1: Pre- and postmortem histopathology. (**A**) Premortem bone marrow aspirate smear demonstrates partially digested red and white blood cells within a bone marrow macrophage (Wright–Giemsa stain, 400×). (**B**) Vertebral bone marrow at autopsy shows notable hemophagocytosis, indicated by black arrow (Hematoxylin and eosin (H&E) stain, 200×). (**C**) Typhus group rickettsia immunohistochemistry (IHC) on postmortem tissue highlights intracellular organisms within splenic endothelial cells (red pigment) (400×, all scale bars = 50 microns). There was a notable absence of inflammatory response to infection in the tissues examined.

On hospital day 5, infectious disease consultation learned the patient had been exposed to feral cats, opossums, and fleas while performing maintenance under the crawl space of his home. Serum for typhus group antibodies via indirect immunofluorescence assay (IFA) was collected, and the patient was started on empiric doxycycline. The bone marrow biopsy eventually demonstrated hypercellular marrow with focal hemophagocytosis (Figure 1). Additionally, hyperferritinemia, hypertriglyceridemia, hypofibrinogenemia, and elevated soluble IL2-R were all noted (Table 1). An H-score (a diagnostic tool developed to estimate the probability of having HLH) was calculated to be 264, indicating a >99% likelihood of HLH. Due to concern for cytokine release syndrome, the patient was given tocilizumab, and he was started on the HLH94 protocol (etoposide and dexamethasone) with rituximab on day 8.

On hospital day 10, the patient died following a cardiopulmonary arrest. Typhus group IFA (IgM and IgG) would return non-reactive for specimens obtained at day 5, but specimens obtained on the day of death and tested by IFA with serial two-fold dilutions would reveal an IgM of 1:512 and an IgG that remained non-reactive.

**Figure 2:**
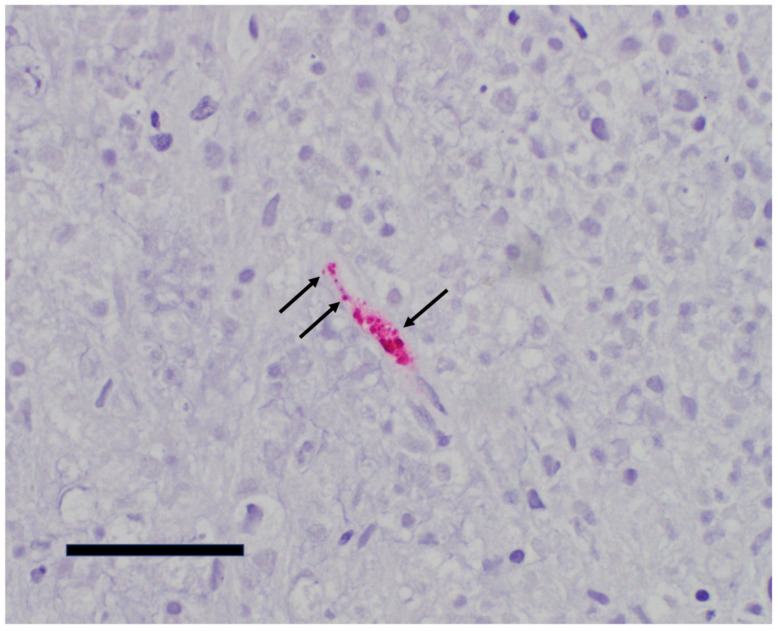
Case 1: Typhus group rickettsia IHC on postmortem tissue highlights intracellular organisms within splenic endothelial cells (red pigment) (400×, scale bar = 50 microns). A few individual rickettsiae are indicated by the arrows.

An autopsy was performed. Pertinent gross findings included an enlarged spleen with multiple reticular discolorations, a markedly yellow-brown liver, and normal-appearing mediastinal, abdominal, and retroperitoneal lymph nodes. Microscopic examination revealed trilineage hematopoiesis with hemophagocytosis within the bone marrow, splenic infarcts, hepatic steatosis with early centrilobular ischemic changes, and diffuse alveolar hemorrhage. Sections of the brain showed clearing of chromatin in astrocyte nuclei in the globus pallidus, substantia nigra, and cerebellar dentate nucleus (Alzheimer type II change, a finding associated with liver disease). The typical histopathological findings associated with severe rickettsiosis were absent, but considering the reactive IgM suggesting murine typhus, immunohistochemistry (IHC) analysis was performed for typhus group rickettsiae using rabbit anti-*R. typhi* polyclonal antibodies diluted at 1:300. Stained intracellular organisms were noted in sections of the lungs, liver, spleen, and brain confirming the diagnosis of typhus group rickettsiosis (Figure 1 and Figure 2).

## 3. Case 2

A 66-year-old male with a history of rheumatoid arthritis presented from a cruise ship infirmary to the emergency department for undifferentiated shock. One week prior the patient developed subjective fever, generalized weakness, and fatigue. Despite persistent symptoms, he boarded a cruise ship destined for the Caribbean, 4 days after symptom onset. During the cruise his fatigue worsened, and he developed lightheadedness leading him to seek medical attention at the ship’s infirmary. Other medical history included chronic kidney disease stage 3a. His home medications included methotrexate and sulfasalazine. Social history included occasional alcohol use (~3 drinks/week) and smoking (unable to determine pack-years). The patient lived in a suburban community in northern Texas. While on the cruise ship the patient was febrile to 38.2 °C, hypotensive, and tachycardic. An EKG revealed atrial fibrillation with rapid ventricular response. While in the ship’s infirmary, the patient was given crystalloid infusion boluses followed by initiation of norepinephrine.

Upon docking, the patient was emergently transferred to our institution. Physical examination at intake demonstrated tachycardia (111 beats/min) with other vital signs within normal limits. Within several hours, however, the patient deteriorated, demonstrating hypotension (85/68 mm of Hg), tachycardia (190 beats/min), tachypnea (40 breaths/min), fever (39.1 °C), and hypoxemia (83% SpO_2_). He appeared diaphoretic with an irregular heart rhythm, and crackles were present throughout all lung fields. Skin examination revealed mottling of his extremities, but no apparent rash. Laboratory testing on admission demonstrated leukocytosis, thrombocytopenia, lactic acidosis, coagulopathy, elevated creatinine, elevated hepatic aminotransferases, and elevated triglycerides (Table 1).

Early in the hospital course, cardioversion was attempted for atrial fibrillation with rapid ventricular response, but this failed. Digoxin was subsequently administered followed by a continuous infusion of amiodarone. The patient was intubated for acute hypoxic respiratory failure and required maximal vasopressor support for refractory hypotension. Meropenem and vancomycin were initiated for septic shock, and doxycycline was added for possible severe rickettsiosis. Serum for typhus group serology was sent. Due to the prolonged activated partial thromboplastin time, thrombocytopenia, hypofibrinogenemia, and risk of bleeding, he was given two units of fresh frozen plasma, two units of cryoprecipitate, and a unit of platelets. CRRT was initiated for AKI. Computed tomography of the chest with pulmonary angiogram demonstrated upper lobe ground glass opacities and small bilateral pleural effusions, and ultrasound of the abdomen revealed hepatomegaly with signs of hepatic steatosis.

**Figure 3:**
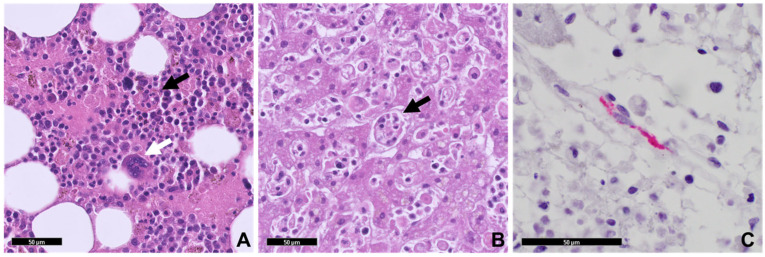
Case 2: Postmortem histopathology. (**A**) Vertebral bone marrow section showed prominent hemophagocytosis. A macrophage with engulfed hematopoietic elements is indicated by black arrow; megakaryocyte is highlighted by white arrow (H&E stain, 200×). (**B**) Liver demonstrated hepatocyte ischemic changes and prominent sinusoidal hemophagocytosis highlighted by arrow (H&E stain, 200×). (**C**) Typhus group rickettsia IHC demonstrates intracellular organisms within splenic endothelial cells (red pigment) (400×, all scale bars = 50 microns). Similar to the first case, the expected inflammatory reaction of severe rickettsiosis was not observed.

**Figure 4:**
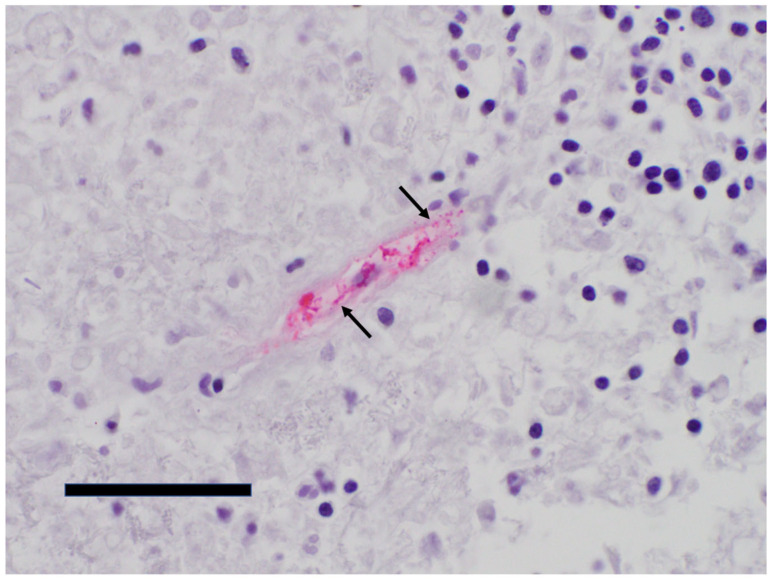
Typhus group rickettsia IHC demonstrates intracellular organisms within splenic endothelial cells (red pigment) (400×, scale bar = 50 microns). Arrows indicate representative examples of individual rickettsiae.

The patient suffered cardiac arrest and died on hospital day 2. His blood cultures would eventually reveal no growth. Typhus group IFA, collected approximately 1 week after onset of symptoms, returned reactive (IgM 1:256 and IgG 1:64). An autopsy was performed. Pertinent gross findings included splenomegaly and enlarged mediastinal lymph nodes. Microscopic examination revealed extensive macrophage activation and marked phagocytosis of red and white blood cells (hemophagocytosis) in the liver, spleen, and bone marrow. Other findings included early hepatic centrilobular ischemia with associated neutrophilic and histiocytic infiltrates within the lobules of the liver, mild hepatic steatosis, mildly congested pulmonary parenchyma, diffuse chronic interstitial inflammation of the lungs, and reactive paracortical hyperplasia in the lymph nodes. Sections from the brain revealed only diffuse vascular congestion. Although there were no microscopic findings consistent with fatal rickettsial illness, IFA serology suggesting murine typhus as an underlying diagnosis prompted further testing by IHC for typhus group rickettsiae. The organisms were identified within endothelial cells of the spleen (Figure 3 and Figure 4), lung, brainstem, and cerebellum, confirming the diagnosis of typhus group rickettsiosis.

## 4. Discussion

HLH is a hyperinflammatory syndrome associated with dysregulation of cytotoxic T lymphocytes, natural killer cells, and macrophages. The resultant activation of these cells causes cytokine storm and multiorgan system immune-mediated injury [13]. HLH can occur as a distinct primary syndrome of immune activation associated with specific genetic causes. Alternatively, it can occur because of immune hyperactivation in the setting of a specific trigger, such as infections, rheumatologic conditions, malignancy, and drug hypersensitivities. It is believed that a genetic predisposition is likely present in those with HLH triggered by these entities [13,14]. Numerous infectious agents have been associated with triggering HLH. The most notable is Epstein–Barr virus, but other viruses (e.g., cytomegalovirus, adenovirus, HIV, parvovirus, rubeola), parasites (e.g., *Leishmania*, *Plasmodium*, *Toxoplasma*, and *Babesia*), fungi (e.g., *Cryptococcus*, *Histoplasma*, *Penicillium*, *Pneumocystis*), and bacteria can also serve as infectious triggers [13].

Historically, the diagnosis of secondary hemophagocytic lymphohistiocytosis (HLH) has relied on the HLH-2004 criteria, which require the presence of five or more of the following features: fever, splenomegaly, bicytopenia, hypertriglyceridemia and/or hypofibrinogenemia, hemophagocytosis on bone marrow biopsy, low or absent natural killer (NK) cell activity, hyperferritinemia, and elevated soluble CD25 [14]. Although these criteria were initially derived from pediatric patients with familial HLH, they have been widely applied to guide the diagnosis of secondary HLH in adults. Complementing this framework, the H-score has emerged as an additional diagnostic tool, notable for its adaptive risk assessment and reliance on commonly available laboratory tests [15]. Both the HLH-2004 criteria and the H-score have undergone validation across multiple external cohorts. In the most recent validation, which included 13 cohorts of critically ill patients, the H-score demonstrated a mean sensitivity of 82.4% and specificity of 87.6% at a cutoff of 169 [16]. Additionally, the HLH-2004 criteria achieved a mean sensitivity of 86.5% and specificity of 86.1%, with the original requirement of five criteria lowered to four to optimize diagnostic accuracy [16]. Since the diagnosis of secondary HLH is often challenging, as there is no single diagnostic test to confirm the diagnosis, both scoring systems can be utilized to enhance diagnostic confidence.

Several rickettsial species and related intracellular bacteria have been implicated as infectious triggers of hemophagocytic lymphohistiocytosis (HLH), including *Rickettsia rickettsii*, *Rickettsia conorii*, *Orientia tsutsugamushi*, *Rickettsia typhi*, *Ehrlichia chaffeensis*, and *Anaplasma phagocytophilum* [17,18,19,20,21,22]. *Orientia tsutsugamushi*, the causative agent of scrub typhus, is the most frequently reported rickettsial cause [23,24]. There is a growing body of evidence that supports murine typhus as a definitive, albeit uncommon, trigger of secondary hemophagocytic lymphohistiocytosis (HLH). A case report and review of the literature by Zamora Gonzalez et al. summarized nine cases of murine typhus complicated by HLH previously reported in the literature [12]. Notably, this group included a case in which histopathology was performed at autopsy, providing definitive evidence of systemic hemophagocytosis [19]. Seven of these had serologically confirmed murine typhus and met at least five HLH-2004 diagnostic criteria [12]. Two cases, originally reported by Walter et al. and Miguélez et al., lacked detailed serologic or HLH-2004 criteria data [25,26]. A serologically confirmed case of murine typhus from Los Angeles County, California documented three HLH-2004 criteria, though the remaining criteria were not specified [11]. Additionally, Mandell et al. recently reported a case involving a 34-year-old male who met five HLH-2004 diagnostic criteria in the setting of murine typhus [27]. With the inclusion of our two cases, the cumulative number of reported cases of murine typhus-associated secondary hemophagocytic lymphohistiocytosis reaches thirteen.

The cases herein presented expand on the growing reports describing HLH triggered by murine typhus with microscopic findings on autopsy that deviate from the typical histopathologic findings of a fatal rickettsiosis. There are few published autopsies in those who died of murine typhus [11,28,29,30], but as with other severe rickettsial infections, such as Rocky Mountain spotted fever and louse-borne epidemic typhus, vascular injury corresponds to the systemic endothelial distribution of rickettsiae. The lymphohistiocytic response manifests microscopically in various organs to cause portal triaditis, interstitial nephritis, myocarditis, meningoencephalitis, and interstitial pneumonitis. In the nervous system, inflammatory nodule-like lesions (typhus nodules) represent foci of lymphocytes, macrophages, plasma cells, and glial cells located around blood vessels [29,31]. Interestingly, the examined tissues of these two individuals with HLH, despite abundant diffuse intracellular infection by IHC, did not demonstrate the histopathologic cellular inflammatory changes associated with a severe rickettsiosis.

It has been proposed that an underlying genetic predisposition to HLH is involved in cases triggered by a secondary process (e.g., drug hypersensitivity, infection, malignancy, or rheumatologic disease) [13]. In predisposed individuals, an infection with a robust production of cytokines is thought to lead to immune dysregulation via unchecked CD8+ T-cell activation and amplification of cytokine signaling, leading to HLH. Animal models and human studies have demonstrated that infection with *R. typhi* results in high levels of IFN-γ as well as other cytokines (e.g., IL-6, IL-8, and IL10) [32,33,34]. The early immune response to *R. typhi* includes its interaction with dendritic cells. Early control of infection is provided by natural killer cells, while adaptive responses are mediated by both CD4+ and CD8+ T lymphocytes [35]. We hypothesize that in those with HLH triggered by *R. typhi*, the high levels of cytokines occurring because of infection have resulted in the aberrant hyperinflammatory response leading to an HLH phenotype, rather than an inflammatory response aimed toward the infecting pathogen.

Both men had possible host risk factors for a severe rickettsial illness. The first man had a history of heavy alcohol consumption, which has been associated with severe presentations of rickettsioses [36,37]. The mechanism to explain heavy alcohol use and severe rickettsial disease is unknown, but chronic alcohol ingestion causes numerous deleterious changes in both innate and adaptive arms of immunity [38,39]. Changes include inhibition of antigen presentation by dendritic cells, the number and function of natural killer cells, and the ability of cytotoxic T cells to expand and respond to pathogens [39,40,41]; these are elements of immunity important to the control of infection with rickettsiae [35]. The second man was on disease-modifying antirheumatic drugs (sulfasalazine and methotrexate) for rheumatoid arthritis. Although these agents are not potent suppressants of the immune system, sulfa-containing drugs have been associated with worsened outcomes when used in those with rickettsioses [42,43].

Following diagnosis, treatment is individualized based on disease extent, duration, and underlying trigger—secondary HLH cannot be treated with a single standardized approach [14,44]. For patients with secondary HLH related to rickettsial disease, treatment is based on the severity of end organ damage as classified by the Histocyte Society. In those with mild disease (no signs of end-organ damage, excluding the coagulation/hematologic system), treatment can be limited to targeted antimicrobial therapy with doxycycline [45]. Corticosteroids can be considered to attenuate the hyperinflammatory response; however, other immunomodulating therapy should be withheld. Patients with moderate and severe HLH, as demonstrated by elevated sequential organ failure assessment (SOFA) scores (≤2 for moderate, ≥3 for severe), should receive corticosteroids in addition to antimicrobials. Intravenous immunoglobulin can be considered (some studies have shown a mortality benefit in patients with infectious triggers of HLH), but further research is needed [46,47]. The HLH-94 treatment protocol (a regimen consisting of etoposide, dexamethasone, and cyclosporin) should generally be avoided in HLH from rickettsial disease [44].

Murine typhus is a burgeoning infectious threat expanding in geographic distribution—especially in California and Texas [48]. Only a small proportion of those with murine typhus develop severe life-threatening manifestations, but as the number of cases increase, so will the cases that develop acute kidney injury, respiratory failure, meningoencephalitis, other sequelae, and death. The growing reports of HLH triggered by typhus group rickettsiae is alarming. Although likely a relatively uncommon event, the two cases recognized in the same year at a single institution, as well as the other case reports published in the literature, support the increasing burden of murine typhus as a cause of febrile illness. The cases described in this report highlight the importance of early recognition and treatment with doxycycline, and they underscore the need to understand the early disease events and pathophysiology that lead to the immune dysregulation that triggers HLH.

## Figures and Tables

**Table 1:** Case 1 and 2 laboratory values just prior to death.

Variable	Reference Range	Case 1	Case 2
Hemoglobin (g/dL)	12.2–16.4 g/dL	9.2 g/dL	15.8 g/dL
White-cell count (per µL)	4.2–10.7 10^3^/μL	14.01 10^3^/μL	24.71 10^3^/μL
Platelet count (per µL)	150–328 10^3^/μL	13 10^3^/μL	35 10^3^/μL
Alanine aminotransferase (U/L)	5–50 U/L	2595 U/L	140 U/L
Aspartate aminotransferase (U/L)	13–40 U/L	>3750 U/L	317 U/L
Alkaline Phosphatase (U/L)	34–122 U/L	137 U/L	180 U/L
Total Bilirubin (mg/dL)	0.1–1.1 mg/dL	12.3 mg/dL	5.9 mg/dL
Lactate Dehydrogenase (U/L)	120–246 U/L	>5000 U/L	636 U/L
Sodium (mmol/L)	135–145 mmol/L	130 mmol/L	129 mmol/L
Urea nitrogen (mg/dL)	7–23 mg/dL	62 mg/dL	79 mg/dL
Creatinine (mg/dL)	0.6–1.25 mg/dL	2.50 mg/dL	3.51 mg/dL
Ferritin (ng/mL)	18–464 ng/mL	>10,000 ng/mL	-
Triglycerides (mg/dL)	30–170 mg/dL	1002 mg/dL	175
Fibrinogen (mg/dL)	167–453 mg/dL	95 mg/dL	97 mg/dL
D-dimer (µg/mL)	<0.5 µg/mL	7.58 µg/mL	76.95 µg/mL
Whole Blood Lactic Acid (mmol/L)	0.5–2.2 mmol/L	5.48 mmol/L	13.44 mmol/L
Soluble IL2R (pg/mL)	175.3–858.2 pg/mL	10,810 pg/mL	-
Typhus Fever Ab, IgM	<1:64	1:512	1:256
Typhus Fever Ab, IgG	<1:64	<1:64	1:64

## Data Availability

The original contributions presented in this study are included in the article. Further inquiries can be directed to the corresponding author(s).
